# Safety of targeting ROR1 for cancer immunotherapy with chimeric antigen receptor-modified T cells in a primate model

**DOI:** 10.1186/2051-1426-2-S3-P3

**Published:** 2014-11-06

**Authors:** S Carolina Berger, Daniel Sommermeyer, Michael Hudecek, Michael Berger, Ashwini Balakrishnan, Paulina Paskiewicz, Paula Kosasih, Christoph Rader, Stanley Riddell

**Affiliations:** 1 Fred Hutchinson Cancer Research Center, University of Washington, Seattle, WA, USA; 2 University of Würzburg, Würzburg, Germany; 3 Technical University of Munich, Munich, Germany; 4 Scripps Florida, The Scripps Research Institute, Jupiter, FL, USA

## Background

Immunotherapy with T cells expressing chimeric antigen receptors (CARs) specific for a tumor cell-surface molecule is effective for CD19^+ ^B cell malignancies. There is interest in extending CAR-T cell therapy to epithelial tumors, which requires identifying molecules that can be targeted safely. The receptor tyrosine kinase-like orphan receptor 1 (ROR1) is expressed at the cell-surface in chronic lymphocytic leukemia, mantle cell lymphoma, and many epithelial malignancies where it contributes to tumor survival. ROR1 is also abundantly expressed in embryogenesis but the cell surface isoform is absent from vital adult tissues by Western blot. However, ROR1 is expressed on pre-B cells and adipocytes, and low levels of transcripts are detected in lung and pancreas, raising concern that targeting ROR1 may cause serious toxicity, as seen in clinical trials of gene-modified T cells for other targets that are not completely tumor-restricted in expression. We developed a CAR (R12) that recognizes a region of ROR1 conserved in macaques and humans but not mice. Here, we transduced autologous T cells from *macaca mulatta *with the R12-CAR and a control vector, and studied their safety, migration, and persistence after adoptive transfer.

## Methods

ROR1-expression was examined by quantitative RT-PCR. Lymphoreplete macaques received T cell infusions consisting of 1-5x10^8^/kg ROR1-CAR and control T cells that each expressed a unique cell surface marker for in vivo-tracking. Clinical/organ toxicity, T cell persistence, and cytokines were monitored. CAR-T cell function was determined by ablation of ROR1^+ ^B cell precursors and response to challenge with autologous ROR1-transfected T cells.

## Results

ROR1-expression was comparable in human and macaque tissues. Macaque ROR1-CAR-T cells were infused without acute toxicity and persisted in the blood for >3 weeks, albeit at lower levels than control T cells administered at the same cell dose. ROR1-CAR-T cells migrated preferentially to bone marrow and lymph nodes and eliminated endogenous ROR1^+ ^B cells, which coincided with transient increases in plasma IFN-g and IL-6. ROR1-CAR-T cells remained functional in vivo as demonstrated by a 7.7-fold increase in number in response to infusion of ROR1^+ ^T cells.

## Conclusion

High doses (5x10^8^/kg) of functional ROR1-CAR-T cells can be adoptively transferred to macaques without acute toxicity supporting targeting ROR1 in human cancers with CAR-T cells. The induction of transgene product-specific immunity limited long-term persistence of CAR-T cells and analysis of late toxicity, however our data illustrates the value of this model for acute safety-studies for candidate targets for CAR-T cells.

